# Adaptive reference ranges: From A to Z

**DOI:** 10.1371/journal.pone.0323133

**Published:** 2025-05-23

**Authors:** Davood Roshan, Kishor Das, Diarmuid Daniels, Charles R. Pedlar, Paul Catterson, John Newell

**Affiliations:** 1 School of Mathematical and Statistical Sciences, University of Galway, Galway, Ireland; 2 CÚRAM, SFI Research Centre for Medical Devices, University of Galway, Galway, Ireland; 3 Insight Centre for Data Analytics, University of Galway, Galway, Ireland; 4 School of Medicine, University of Galway, Ireland; 5 Faculty of Sport, Technology and Health Sciences, St Mary’s University Twickenham, London, United Kingdom; 6 Orreco Ltd, Business Innovation Unit, University of Galway, Ireland; 7 Institute of Sport, Exercise and Health, Division of Surgery and Interventional Science, UCL, London, United Kingdom; 8 Newcastle United Football Club, United Kingdom; Universita degli Studi di Siena, ITALY

## Abstract

Clinical reference ranges are fundamental in medical diagnostics, offering critical benchmarks for interpreting laboratory test results. Adaptive reference ranges, in particular, are essential for personalised monitoring, as they enable the detection of abnormal values by accounting for individual variability over time. This paper compares two key approaches for generating adaptive reference ranges: the Z-score method and the linear mixed-effects modelling framework. Through simulation studies and real data applications, we provide practical insights into selecting the most appropriate methods for adaptive monitoring in personalised medicine and sport science. Our findings highlight the trade-offs between these approaches, with the Z-score method favouring specificity, while the linear mixed-effects model prioritises sensitivity and offers greater flexibility by incorporating population-level data, accommodating covariates, and effectively handling missing data.

## Introduction

Clinical reference ranges are critical tools in medical diagnostics, serving as benchmarks for interpreting laboratory test results. Traditionally, these ranges are calculated using population-based data and tolerance intervals, which aim to capture a certain proportion of the population’s values (e.g., 95%) with a specified level of confidence (e.g., 90%) for a particular biomarker [1]. While widely used, this method often fails to account for individual variability and temporal changes within a single patient, especially in the context of longitudinal monitoring, leading to potential false positives or false negatives in diagnosis.

This limitation is particularly evident in sports science, where advancements in wearable technologies, such as heart rate monitors, GPS trackers, and accelerometers, often use population-based reference ranges to monitor athletes’ performance and physiological status over time. While these ranges can be useful when only a single measurement is available, they do not accurately reflect an athlete’s performance or physiological status when tracking changes over time, potentially leading to inappropriate training decisions and an increased risk of injury [2].

A more meaningful approach, however, considers an individual’s current data with both population and their own historical data. Such an approach is adaptive reference ranges, i.e. reference ranges that adapt successively as new data are recorded for an individual, enabling more personalised and effective medical care [2, 3].

Early work in adaptive reference ranges can be traced back to Sharpe *et al*.’s [4] study on detecting erythropoietin abuse in athletes. Sharpe *et al*. introduced a Z-score approach that relied on the Normal distribution and unbiased estimates of the mean and variance of previous observations to generate adaptive reference ranges. This approach might be limited though when the number of observations from a given subject is small, i.e. less than six [4]. To address this issue, Sharpe *et al*. proposed to assume a fixed within-subject variability across all individuals so that the method could still be applied to individuals with limited observations. Sottas *et al*. [3] later argued that Sharpe’s Z-score method is unsuitable as the assumption of a fixed within subject variability may not be realistic for many biomarkers. Furthermore, in a more recent study, Sauliere *et al*. [5] highlighted that the correct distribution to use for the Z-score in small sample sizes is the t-distribution. Among the three existing Z-score-based methods for generating adaptive reference ranges, the Z-score method in conjunction with the t-distribution appears to be more broadly applicable across various scenarios and is therefore considered in greater detail in this article. Nevertheless, it should be noted that this method relies solely on within-subject variability and does not account for between-subject variability or any other population-based information, which may also limit its effectiveness.

Recent studies by Roshan *et al*. [2] and Sottas *et al*. [3] have developed methods for generating adaptive reference ranges that not only account for the variability within an individual’s measurements by incorporating their previous readings but also consider variability across subjects using population-based information. To this end, Sottas *et al*. [3] proposed a Bayesian framework that combines individual measurements with prior population data. This approach not only provides a more accurate quantification of an individual’s characteristics but also allows the reference ranges to adapt as additional data is collected for the individual.

Roshan *et al*. [2] expanded on Sottas’ work by proposing a Bayesian framework that accounts for differing within-individual variabilities, a more realistic assumption that enhances its practical applicability. Additionally, Roshan *et al*. [2] introduced a linear mixed-effects modelling (LMM) framework, which, while maintaining the accuracy of the Bayesian approach, is computationally more efficient and practical for real-world applications. This approach relies on a few summary statistics at the individual level, combining all the necessary information from previous observations to build reference ranges.

The Z-score method in conjunction with the t-distribution, while very simple, may sacrifice some sensitivity or specificity compared to approaches that incorporate broader population-based data. Despite this, the use of the Z-score approach remains prevalent due to its simplicity and widespread familiarity in various applications. Sottas *et al*. [3] demonstrated through a real case study how the Bayesian approach outperforms the Z-score method with Normal distribution in terms of false positive rate when detecting abnormal values in longitudinal testosterone/epitestosterone (T/E) ratio data used in anti-doping tests. Additionally, Roshan *et al*. [2] showed through a simulation study that, despite a negligible difference in performance between the LMM framework and the Bayesian approach, the former outperforms the latter in terms of computational time.

However, despite these comparisons, a direct evaluation of the LMM framework against the Z-score method in conjunction with the t-distribution, hereafter referred to as Z-score method, has not been thoroughly explored. This comparison is crucial, as it would provide a comprehensive evaluation of these methods, helping to determine the most suitable approach for various practical applications. This is particularly important in scenarios where accuracy and computational efficiency (a challenge often faced by the Bayesian method) are both critical.

Therefore, this paper aims to compare the performance of the mixed-effect modelling framework with the Z-score approach, providing guidelines for when each method can be safely used in practice. By evaluating these methods, we hope to offer a clearer understanding of their applications and limitations, ultimately improving the accuracy and reliability of clinical reference ranges in personalised medicine. To achieve this, a thorough simulation study alongside real-world data application will be provided to evaluate the effectiveness of each method in identifying abnormal values during longitudinal monitoring. The comparison will primarily focus on sensitivity and specificity as performance metrics, where sensitivity refers to the percentage of correctly identified abnormal observations, while specificity indicates the percentage of correctly identified normal observations.

The rest of the paper proceeds as follows: In the Methods section, methods for generating adaptive reference ranges using both the Z-score and mixed-effect modelling frameworks will be reviewed. The Simulation Study section presents the results of a simulation study to assess the performance of these methods. In the Real Data Application section, we apply the methods to real data collected from athletes in elite sports, and finally, the Discussion and Conclusion sections offer insights and closing remarks.

## Methods

Let $Y_{1},Y_{2},\ldots ,Y_{n}$ are *n* independent measurements of a biomarker from the same subject, where $Y_{j}\sim N(\mu ,\sigma^{2});j=1,2,\ldots ,n$. The new observation, *Y*_*n* + 1_, collected from the same subject is considered to be abnormal if it falls outside of the (1−α)% percentile range of the condi-tional distribution $p(Y_{n+1}|Y_{1},Y_{2},\ldots ,Y_{n})$, where α is the level of significance and 0≤α≤1. This percentile range is called adaptive reference range as the reference ranges are adaptive by taking account of previous readings to progressively learn the characteristics of the individual [2]. In the remainder of this section, a brief discussion on the generation of adaptive reference ranges using the Z-score method and the LMM framework will be given. The Z-score method will be introduced in the *Method 1* section, while the LMM framework will be discussed in the *Method 2* section.

### Method 1

For a new observation *Y*_*n* + 1_ collected from a subject, the following quantity can be calculated [5]:

$$T_{n+1}=\frac{Y_{n+1}-Y_{n}}{\hat{\sigma_{n}}\sqrt{1+\frac{1}{n}}}\sim T^{n-1}$$ (1)

where *T*_*n* + 1_ is the Z-score obtained for the measurement $Y_{n+1},Y_{n}$ and ${\hat{\sigma }}_{n}$ are the sample mean and standard deviation, respectively, obtained from the first *n* measurements from the same subject, ${\text{T}}^{\text{n}-1}$ is a Student T distribution with *n*–1 degrees of freedom.

Equation 1 assumes that all the $Y_{j};j=1,2,\ldots ,n$ are independent to each other. If *T*_*n* + 1_ falls outside of the (1−α)% range of a Student T distribution with *n*–1 degrees of freedom then the observation *Y*_*n* + 1_ will be considered as abnormal.

An adaptive reference range for observation *Y*_*n* + 1_ based on the equation( 1) can be obtained as follows:

$$\mathrm{\backslash Bar}Y_{n}\pm T_{\left(1-\alpha /2\right)}^{n-1}{\hat{\sigma }}_{n}\sqrt{1+\frac{1}{n}}$$ (2)

where, $T_{\left(1-\alpha /2\right)}^{n-1}$ is the (1-α/2)^th^quantile of a t-distribution with (*n*–1) degrees of freedom.

### Method 2

The Z-score method discussed in previous section only takes into account the within subject variability of the individual and do not consider the between subject variability or any other population-based information. An LMM, which takes into account both between- and within-subject variabilities in the biomarker values, can be defined to generate adaptive reference ranges as follows [2, 6]:

$$Y_{ij}=\mu_{i}+\epsilon_{ij};\;\;i=1,2,\ldots ,I;j=1,2,\ldots ,n_{i}$$ (3)

where *Y*_*ij*_ is the ${\text{j}}^{\text{th}}$ measurement from the ${\text{i}}^{\text{th}}$ subject which follows $N(\mu_{i},\sigma_{i}^{2})$, where, $\mu_{i},$ is the random intercept terms for the ${\text{i}}^{\text{th}}$ subject which follows $N(\mu ,\tau^{2})$. Here μ is the population average mean of the biomarker, $\tau^{2}$ is the between subject variability, and $\sigma_{i}^{2}$ is the within subject variability for the ${\text{i}}^{\text{th}}$ subject.

Measurement *j* from subject *i*, *y*_*ij*_, will be considered atypical if it falls beyond the (1−α)% percentile range of $P(y_{ij}|y)$; where *y* refers to both subject *i* measurements prior to time *j* (i.e., $y_{i1},\ldots ,y_{i(j-1)}$) and other historical information from other individuals (i.e., $y_{i^{\prime }j}$; $i^{\prime }\neq i$ and $j=1,\ldots ,n_{i}$). An approximate expectation maximisation algorithm (EM) [7, 8] has been proposed to estimate the model parameters to calculate the following 100*(1−α)% percentile range of the distribution of the future observation, i.e. $P(y_{ij}|y)$:

$$\frac{(\hat{\mu }/{\hat{\tau }}^{2})+\left(\left[(j-1){\bar{y}}_{i}^{\left(j-1\right)}\right]/{\hat{\sigma }}_{i}^{2}\right)}{\left(1/{\hat{\tau }}^{2})+[\left(j-1\right)/{\hat{\sigma }}_{i}^{2}\right]}\pm z_{1-\frac{\alpha }{2}}\sqrt{\frac{1}{\left(1/{\hat{\tau }}^{2})+[\left(j-1\right)/{\hat{\sigma }}_{i}^{2}\right]}+{\hat{\sigma }}_{i}^{2}}$$ (4)

In this section, two methods for generating adaptive reference ranges were introduced. The subsequent sections will explore the effectiveness of these methods by comparing their sensitivity and specificity, utilising both a simulation study and a real data example. All analyses were conducted using R Programming Language (version 4.3.1).

## Simulation study

A simulation study will be carried out in this section to compare the specificity (true negative rate) of the Z-score method and the LMM framework to generate adaptive reference ranges. Assessment of the performance of these methods will be based on the false positive rate (1 – specificity).

The dataset will be generated by simulating values from a normal distribution, adhering to the following statistical model.

$$Y_{ij}=\mu_{i}+\epsilon_{ij}$$ (5)

$$i=1,2,\ldots ,n;j=1,2,\ldots ,n_{i}$$

$$\mu_{i}\sim N(\mu ,\tau^{2});\epsilon_{ij}\sim N(0,\sigma_{i}^{2})$$

where *Y*_*ij*_ is the $j^{\text{th}}$ measurement of the biomarker from the ${\text{i}}^{\text{th}}$ subject, $\mu_{i}$ is the subject specific mean for the ${\text{i}}^{\text{th}}$ subject, $\epsilon_{ij}$ is the measurement error for the measurement *Y*_*ij*_, μ is the population mean, $\tau^{2}$ is the between subject variability and $\sigma_{i}^{2}$ is the within subject variability for the $i^{\text{th}}$ subject.

Based on the data generation model outlined above, the detail of the simulation strategy to evaluate the performance of both the methods under consideration will be provided in the next section.

### Simulation strategy

The effectiveness of various methods for generating adaptive reference ranges will be assessed by determining the false positive rate, which is the percentage of normal observations incorrectly classified as abnormal, at a 5% significance level. It has been noted that the ratio of within-subject variability to between-subject variability can affect the sensitivity and specificity of these methods [2]. Consequently, different scenarios were explored based on varying ratios of within-subject to between-subject variability. Ratios from 0.001 to 5 were considered informed by the real hrCRP dataset used in this article as well as the real data and simulation study presented elsewhere [2]. A total of thirty-six scenarios were examined, with between-subject standard deviations set at $\tau^{2}=0.5,1,\;\&\;5$, and within-subject standard deviations ranging from 0.0005 to 10, maintaining ratios from 0.001 to 5 (Table 1). In addition a population mean of μ=5 was considered as per the requirement of the generating model outlined in the previous section.

**Table 1:** Within-subject standard deviations (cell entries) calculated for 36 different scenarios in the simulation study. The values are obtained by multiplying fixed ratios of within-subject to between-subject standard deviation (rows) with different between-subject standard deviations (columns).

Ratio	Between-subject SD		
	0.5	1	2
0.001	0.0005	0.001	0.002
0.005	0.0025	0.005	0.010
0.010	0.0050	0.010	0.020
0.025	0.0125	0.025	0.050
0.050	0.0250	0.050	0.100
0.075	0.0375	0.075	0.150
0.100	0.0500	0.100	0.200
0.250	0.1250	0.250	0.500
0.500	0.2500	0.500	1.000
1.000	0.5000	1.000	2.000
2.000	1.0000	2.000	4.000
5.000	2.5000	5.000	10.000

In each of the 36 scenarios, 1,000 subjects were generated, leading to a total of N=36,000 subjects in the population, with each subject having *n*_*i*_ = 100 independent measurements. From each scenario, a random sample of 100 subjects was drawn, resulting in a total sample of n=3,600 subjects. The comparison of the methods were made based on these samples. It is important to note that the LMM framework requires a healthy (i.e., control) population to initialise estimates of within- and between-subject variabilities, so a random sample of 120 subjects was also selected from the simulated population.

The false positive rate for the two methods of generating adaptive reference ranges was determined for each subject. Specifically, for a given method and subject, the false positive rate was calculated as the percentage of observations that fell outside the generated adaptive reference ranges. The reference range using the Z-score method can only be calculated starting from the third observation, as the first two observations are needed to estimate within-subject variability. Consequently, comparisons of false positive rates for the two methods were made only from the third observation onward.

Having established the simulation strategy and parameters, the results from these simulations will be presented, highlighting the performance and implications of the different scenarios analysed, in the next section.

### Simulation results

**Fig 1:**
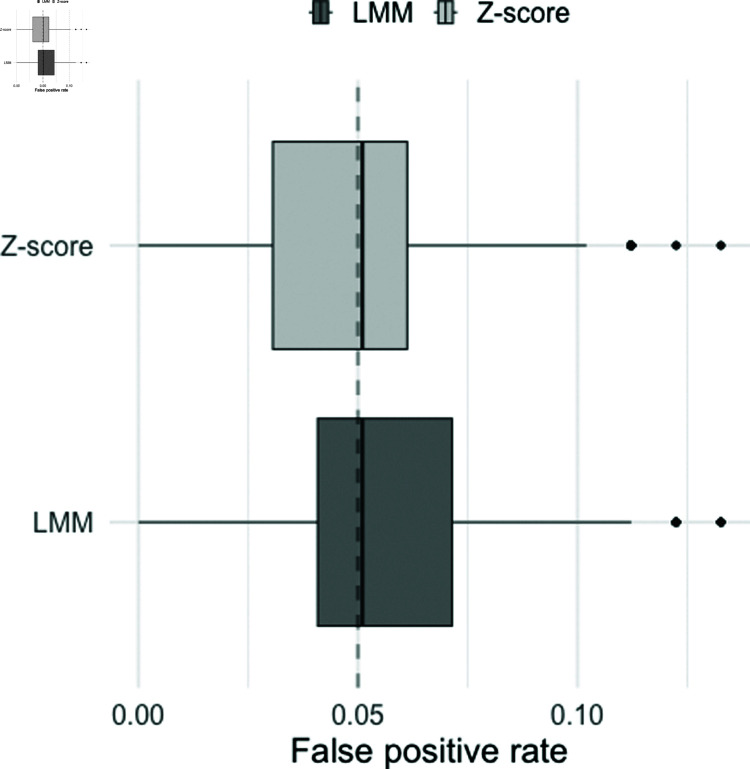
Distribution of the overall false positive rates for Z-score method and the linear mixed-effects modelling (LMM) framework. The distribution was obtained based on a sample of 3,600 subjects drawn from a simulated population of 36,000 subjects. The dashed line indicates the nominal level of significance (0.05).

Fig 1 illustrates a boxplot of the false positive rates obtained from 3,600 different subjects sampled from the simulated population. It is apparent that the median false positive rates for both methods (0.051) match the nominal significance level (α=0.05), suggesting that both methods exhibit similar median specificity. However, the distribution of false positive rates differs slightly between the methods. While the median false positive rates are identical, the Z-score method shows a left-skewed distribution, whereas the LMM framework displays a right-skewed distribution. This result suggests that the LMM framework tends to yield a slightly higher false positive rate compared to the Z-score method.

**Fig 2:**
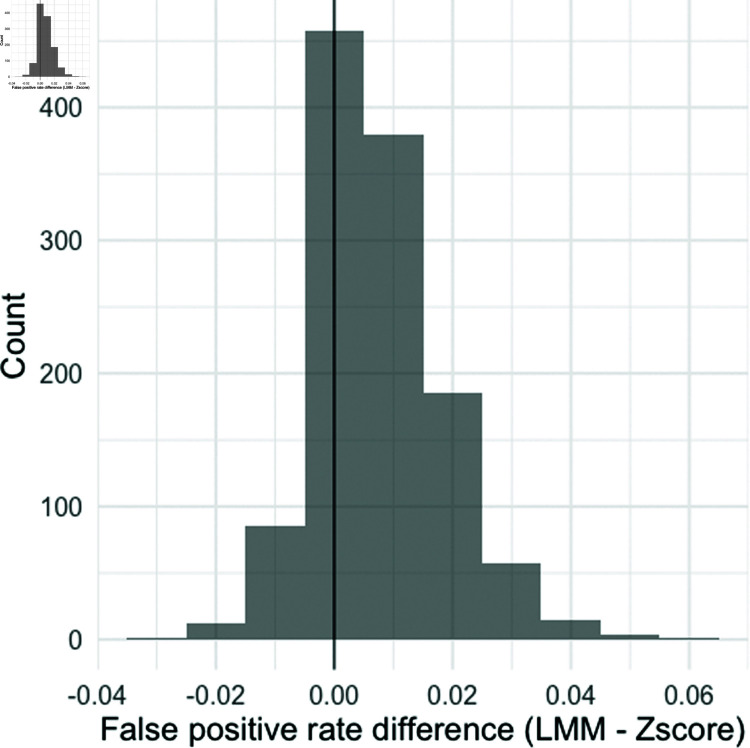
Histogram of the pairwise differences in false positive rates between the linear mixed-effects modeling (LMM) framework and the Z-score method. The histogram was obtained from a sample of 3600 from the simulated population. The black vertical line indicates zero (i.e. no difference in false positive rates between the two methods).

Fig 2 shows the histogram of the pairwise difference between false positive rate by the Z-score method and the LMM framework. The result shows that the false positive rate of the LMM framework is consistently higher compared to the Z-score method for most of the subjects.

**Fig 3:**
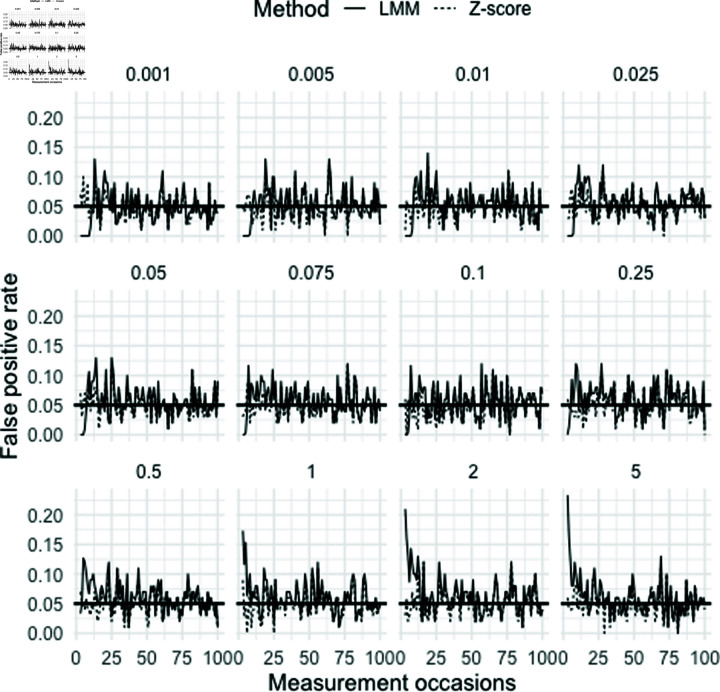
False positive rate over different measurement occasions. False positive rates calculated at each measurement point for the Z-score method and the linear mixed-effects modelling (LMM) framework for various ratios of within- and between-subject variability. The horizontal solid black line represents the nominal significance level (0.05).

To further explore the differences in false positive rates shown in Fig 1, Fig 3 displays the false positive rates calculated at each measurement point for the two methods. This provides a more detailed comparison of how the false positive rates change over time for each method. The observed differences in false positive rates between the two methods appear to be due to the difference in false positive rate in the early measurements. Depending on the ratio of within- to between-subject variability, the LMM framework sometimes results in a lower false positive rate and sometimes a higher one compared to the Z-score method. However, as more measurements are collected, the false positive rates for both methods become similar by the $30^{\text{th}}$ measurement point. Beyond this point, the two methods exhibit relatively stable false positive rates, indicating that with enough measurements, the performance of all methods converges to the nominal significance level (α=0.05) as intended.

In this section, we conducted a simulation study to compare the specificity of adaptive reference ranges generated by both the methods. However, sensitivity analysis was not performed due to the inherent challenges in simulating true abnormal values, making it difficult to generate reliable true positive cases. Therefore, the subsequent section will explore both sensitivity and specificity using real data applications, where true abnormalities can be observed and analysed.

## Real data application

In this section, we aim to explore the application of adaptive reference ranges in English Premier League (EPL) soccer players using a Point-of-Care (POC) testing of high-sensitivity C-Reactive Protein (hsCRP) biomarker. The biomarker hsCRP is a well-established marker of inflammation and has been shown to be a sensitive indicator of inflammatory response following a soccer match [9].

Data were collected from four first-team EPL players (age: 22.9 ± 2.3 years, height: 178.8 ± 7.1 cm, weight: 73.9 ± 6 kg, and BMI: 23.1 ± 0.75) between 10/08/2019 and 16/12/2019. Testing took place between 9:00 a.m. and 11:00 a.m., with pre-game high-sensitivity C-Reactive Protein (hsCRP) levels (CRPpre) measured one day before the match (Match Day -1) and post-match hsCRP levels (CRPpost) assessed on either one, two, or three days after the match (Match Day +1, +2, or +3), or a combination of these days. Subjective well-being evaluations were conducted before each blood sampling. Ethical approval was granted by the University of Galway research ethics committee, and all participants provided written informed consent.

For the LMM framework, a “leave-one-athlete-out” strategy was utilised to generate adaptive reference ranges. In this approach, reference ranges for each athlete were constructed by re-estimating all relevant parameters while excluding that athlete’s data, thereby simulating a scenario if all readings from the individual were new values to assess. Data points were omitted when generating reference ranges if athletes reported symptoms of illness, injury, or significantly high levels of fatigue and soreness to the research personnel or club medical team. Additionally, players were classified as injured or ill by the medical staff when appropriate.

The adaptive reference ranges then classify an athlete’s hsCRP values as either normal or abnormal, depending on whether they fall within or outside the established reference range at each time point. To assess the effectiveness of these reference ranges in detecting abnormal hsCRP values, sensitivity and false positive rate (1-specificity) were calculated at different significance levels.

**Fig 4:**
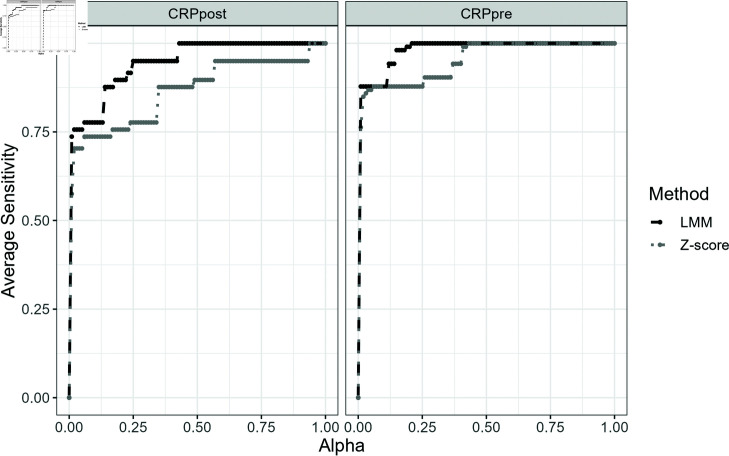
Average Sensitivity vs. Significance Level (Alpha) for post-match (CRPpost) and pre-match (CRPpre) measurements of high-sensitivity C-Reactive Protein (hsCRP). The plot compares the Z Score method (dotted line) with the linear mixed-effects model (LMM) (dashed line).

**Fig 5:**
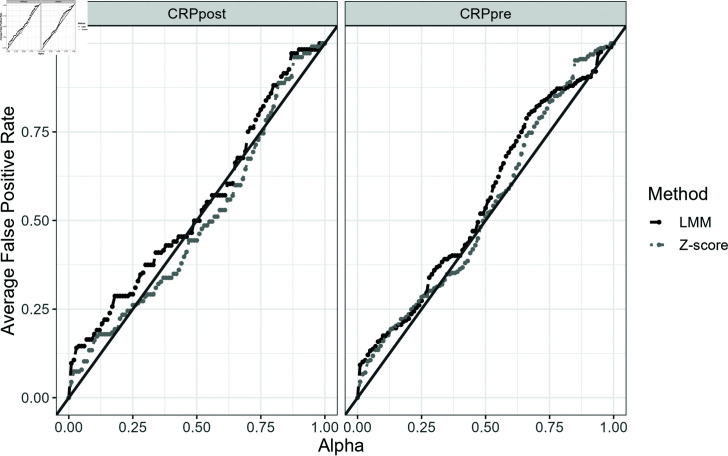
Average False Positive Rate (1 – Specificity) vs. Significance Level (Alpha) for post-match (CRPpost) and pre-match (CRPpre) measurements of high-sensitivity C-Reactive Protein (hsCRP). The plot compares the Z-Score method (dotted line) and the Linear Mixed Effect model (LMM) (dashed line) against the line of equality (solid line).

Figs 4 and 5 displays the performance of the LMM framework and Z-score in terms of their average sensitivity and specificity across different significance levels.

From the Fig 4, we can observe that the sensitivity for both methods is generally high across most significance levels, approaching or reaching 1.0, indicating excellent detection of true abnormal hsCRPs. For both CRPpost and CRPpre, the Z-score method shows lower sensitivity at certain significance levels, while the mixed effect modelling framework consistently maintains a higher sensitivity across the range. This suggests that the LMM framework method is better at identifying true abnormal values in both pre- and post-match measurements.

Fig 5 shows that both the LMM framework and the Z-score methods show varying levels of performance in terms of false positive rate. Overall, the Z-score method aligns more closely with the line of equality, indicating a better consistency between false positive rate and significance levels compared to the LMM framework, suggesting that it achieves a more balanced performance with fewer false positives.

**Fig 6:**
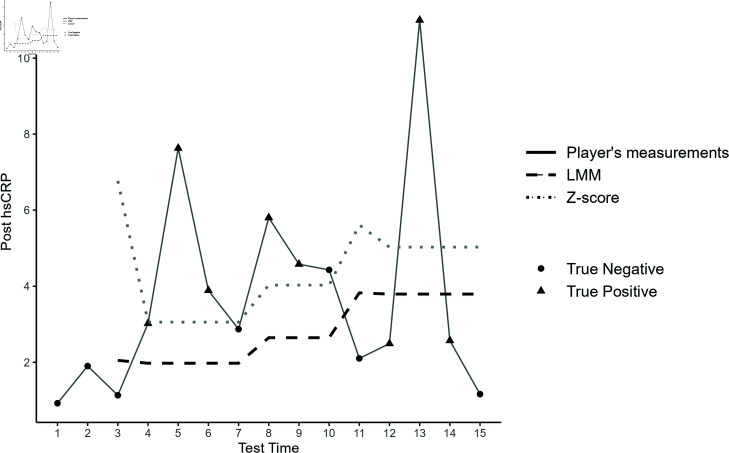
Adaptive reference ranges of post hsCRP for a particular player. Fifteen longitudinal post hsCRP measurements (solid line) for a specific player, with circles representing true negative (normal) values and triangles indicating true positive (abnormal) values. Adaptive reference ranges are shown using LMM (dashed line) and Z-score (dotted line) methods.

Fig 6 illustrates the longitudinal post-hsCRP measurements for a single player, comparing the adaptive reference ranges generated by the Z-score and LMM methods. Given that low hsCRP values are not clinically significant, we focused primarily on upper-sided adaptive reference ranges, with the lower limit set to 0. As can be seen, the Z-score method, influenced by highly variable initial measurements, produces wider reference ranges, leading to missed true positives at time points 4, 12, and 14, while correctly identifying true positives at time points 5, 6, 8, 9, and 13, resulting in a sensitivity of 0.625 (5 out of 8). In contrast, the LMM method generates narrower and more precise reference ranges. This approach successfully flags true positives at time points 4, 5, 6, 8, 9, and 13, achieving a higher sensitivity of 0.75 (6 out of 8), though it also fails to capture abnormalities at time points 12 and 14. It is interesting to see that, the LMM method identifies two false positives at time points 7 and 10, compared to only one false positive for the Z-score method at time 10. Notably, these false positives identified by the LMM approach occurred either just before or immediately after actual abnormal values, suggesting that LMM-derived reference ranges could serve as an effective early warning system. Alternatively, these results might indicate incomplete recovery in the player, as their hsCRP values still remained outside their personalised reference ranges. In contrast, the Z-score method consistently produces wider reference ranges, failing to capture such early warnings and missing potential signs of incomplete recovery or elevated risk.

In summary, while the LMM framework has lower specificity compared to the Z-score, it demonstrates higher sensitivity. This indicates that the mixed effect modelling framework could be better at correctly identifying true abnormal values, even though it results in a slightly higher false positive rate. In the context of sport science and other applied settings, prioritising sensitivity over specificity can be crucial, as failing to identify an at-risk or injured player (false negative) may lead to negative outcomes, such as worsening the injury or leading to long-term health issues. Conversely, while a higher false positive rate (incorrectly flagging a healthy player) might seem undesirable, these alerts can still be valuable. They could indicate potential early signs of elevated risk, even if no current injury or illness is present. Thus, using a method which prioritises sensitivity, can help managers and coaching staff take precautionary measures, such as reducing training load or offering additional recovery time, ultimately serving as a proactive approach to injury prevention.

## Discussion

In this paper, methods for developing adaptive reference ranges were reviewed and discussed from two perspectives: (i) relying solely on within-subject variability, based only on the subject’s past measurements, and (ii) incorporating both within-subject variability (from the individual’s past measurements) and between-subject variability (derived from other individuals in the study). For the first perspective, the Z-score method in conjunction with the t-distribution [5] was identified and presented as a more suitable alternative to the Z-score method proposed by Sharpe *et al*. [4], addressing the limitations of normality assumptions in small sample size scenarios. For the second perspective, an LMM framework was presented as a practical alternative to the Bayesian method.

It should be noted that both methods assume that measurements from a given subject are independent of each other and normally distributed. However, the within-subject variability and the mean of the normal distribution may vary between subjects, as described in equation (3). For non-normally distributed data, a transformation to normality can be applied to meet this assumption.

Between the two methods discussed, a negligible difference was observed in the false positive rates that is mainly due to the wider adaptive reference ranges produced by Z-score compared to the LMM and particularly for the first 20-30 observations. The narrower reference ranges produced by LMM are therefore more sensitive to small changes and as a result produce more false positive rates. However, this could be considered as an advantage of LMM as not only it would be more sensitive to individual measurement variability, providing a more robust analysis in the presence of outliers but also it could be considered as an early warning system that warrant further attention and review.

Moreover, while the Z-score method fails to account for between-subject variability, the LMM framework explicitly models this, leading to better performance in detecting true anomalies. This is mainly because Z-score does not rely on any extra population or samples from the other individuals in generating adaptive reference ranges indicating that some sensitivity might be lost when compared to a method that does incorporate such information [3]. This limitation is particularly relevant in sports science and in anti-doping regulations. For example, using only individuals’ previous records to assess the new test result will lead to a significant decrease in the sensitivity if the person initiated doping before data collection.

Another advantage of the LMM framework is its flexibility in incorporating covariates and handling missing values, as well as its generalisability to multivariate scenarios where multiple outcomes are collected from individuals over time. This approach is particularly useful when the goal is to determine whether an individual’s joint measurements are atypical at a given time, based on both the individual’s historical data and the population-level data while accounting for other covariates.

## Conclusions

This paper demonstrates that while the Z-score approach is straightforward and widely used, it falls short in applications where between-subject variability and covariates play a significant role. The mixed effect modelling framework, by addressing these limitations, provides a more reliable tool for longitudinal biomarker monitoring and abnormality detection.
